# Salt Tolerance of Sea Flax (*Linum maritimum* L.), a Rare Species with Conservation Interest in Eastern Spain

**DOI:** 10.3390/plants13020305

**Published:** 2024-01-19

**Authors:** Diana M. Mircea, P. Pablo Ferrer-Gallego, Inmaculada Ferrando-Pardo, Oscar Vicente, Ricardo Mir, Monica Boscaiu

**Affiliations:** 1 Mediterranean Agroforestry Institute (IAM), Universitat Politècnica de València, Camino de Vera s/n, 46022 Valencia, Spain; dmircea@doctor.upv.es; 2 Servicio de Vida Silvestre y Natura 2000, Generalitat Valenciana, Avda Comarques del País Valencia, 114, Quart de Poblet, 46930 Valencia, Spain; flora.cief@gva.es (P.P.F.-G.); endemica_cief@gva.es (I.F.-P.); 3 Institute for the Conservation and Improvement of Valencian Agrodiversity (COMAV), Universitat Politècnica de València, Camino de Vera s/n, 46022 Valencia, Spain; ovicente@upvnet.upv.es (O.V.); rimimo@upvnet.upv.es (R.M.)

**Keywords:** salt stress, halophytes, soil analysis, plant growth analysis, biochemical parameters, biodiversity, conservation programmes

## Abstract

Seldom found in saltmarshes, *Linum maritimum* is a halophyte of great conservation interest in the eastern Iberian Peninsula. Although the species has been reported in different plant communities, there is no information on its range of salinity tolerance or mechanisms of response to environmental stress factors. In this study, *L. maritimum* plants were subjected to increasing salt concentrations in controlled conditions in a greenhouse. After six months of watering with salt solutions, only plants from the control, 50 mM and 100 mM NaCl treatment groups survived, but seeds were produced only in the first two. Significant differences were found between the plants from the various treatment groups in terms of their growth parameters, such as plant height, fresh weight, and the quantity of flowers and fruits. The main mechanism of salt tolerance is probably related to the species’ ability to activate K^+^ uptake and transport to shoots to partly counteract the accumulation of toxic Na^+^ ions. A biochemical analysis showed significant increases in glycine betaine, flavonoids and total phenolic compounds, highlighting the importance of osmotic regulation and antioxidant compounds in the salt tolerance of *Linum maritimum*. These findings have implications for the conservation of the species, especially under changing climatic conditions that may lead to increased soil salinity in its Mediterranean distribution area.

## 1. Introduction

Climate change is an increasingly critical problem with far-reaching implications for the Earth’s ecosystems and the species they harbour. Rising global temperatures and climate instability [1] are causing temperature fluctuations, changes in precipitation patterns and an increase in the frequency of severe weather events at an unprecedented rate. These environmental changes significantly affect plants, especially those that have evolved in specific habitats, such as salt marshes.

Many salt marshes have been reduced to a small fringe along estuary borders or wholly destroyed due to enclosure for agricultural use, urbanisation or the construction of ports and other industrial or touristic infrastructures. Salt marshes host a variety of specialised plant communities, which are frequently of significant conservation concern [2].

Nowadays, it is commonly acknowledged that salt marshes are essential for wildlife protection, coastal defence and as a significant supply of nutrients and organic matter for a variety of species [3]. The most significant environmental stressor in such habitats is the soil salinity, which restricts the presence of plant species. Halophytes are the only plants that have developed special defences against excessive salinity and even thrive in salinised habitats [4].

High soil salinity causes salt stress, a primary abiotic factor influencing plant growth and development [5,6]. Halophytes possess salt tolerance mechanisms and have evolved an array of biochemical responses to cope with the challenges posed by the high salt concentrations in their habitats [7].

Understanding the genetic, biochemical and physiological basis of salt tolerance in such species is essential, as this could help develop crop cultivars that are more tolerant to changing environmental conditions [8,9] and also aid conservation programmes for rare and endangered halophyte taxa that may be threatened by future environmental changes triggered by global warming.

One aspect that is seldom considered in the analysis of the salt tolerance of plants is their reproductive biology, although flowering and fruiting patterns are essential for their adaptation to salt-affected environments [10,11]. Flowering is one of the most critical phases of the plant life cycle, which responds to altering conditions, including temperature and photoperiod. These signals, which are influenced by climate change, affect the timing of flowering in many different species [12,13]. The consequences of these shifts in flowering patterns for plant populations and ecosystems are a subject of increasing concern [14]. However, only a few studies have analysed the effect of salinity on the reproductive traits of halophytes, except germination, which has been extensively studied in numerous salt-tolerant species [15,16,17,18].

The family Linaceae comprises 22 genera [19] and approximately 300 species [20,21,22,23]. The most diverse genus in this family is *Linum* L., with about 230 species distributed in the temperate regions of the world (Mediterranean region, southern North America, Mexico, and South America) [24,25,26,27]. The Mediterranean area is one of the main centres of diversity for this genus and harbours about 75 species [28]. The genus has economic importance, especially the common flax, *L*. *usitatissimum* L., cultivated for its seeds (flax oil) and fibre production [29,30].

This study is focused on an endemic halophyte, *Linum maritimum* L., generally known as sea flax, which is an iconic coastal plant species that faces the relentless pressures of salt stress due to its presence in salt marshes, dunes and other saline environments [2,31,32].

*Linum maritimum* is distributed throughout the Mediterranean basin, without a UICN category but with interest in La Albufera Natural Park (Valencian Community, Spain), the most relevant protected area of the Valencian Community, where its populations are scarce [32,33,34,35,36]. It is a perennial plant that is 15–60 cm tall with a branching woody layer. Yellow blooms produce a corymbiform panicle in groups and sepals that match the globular capsule. The seeds are flat, ellipsoidal and dispersed by barochory [26,37]. It is a hemicryptophyte that blooms from May to August, and its fruiting season is from July to October [35,37]. Its distribution is from the Mediterranean region and South Europe (Spain (incl. Balearic Islands), Portugal, France, Italy, Austria, Albania, Greece, Turkey, Cyprus, Croatia, Slovenia and Palestine) to North Africa (Morocco and Algeria) [28,31,38]. It is present on some islands such as Mallorca, Corsica, Sardinia or Cyprus, overcoming obstacles, such as extreme salinity, a shortage of water, and repeated exposure to salty winds [39]. *Linum maritimum* is not included in any protection catalogue in Spain; however, its habitats are threatened by land degradation, agricultural and urban development, changes in the soil regime due to floods and increased salt levels, soil over-fertilisation, competition with invasive plant species, wildfires and touristic pressure. On the other hand, *L. maritimum* is a characteristic and bioindicative species of the quality of the vegetation in saline environments, especially in coastal salt marshes. Together with common structural halophytes, other salt-tolerant species, less frequent or even rare, are precisely those on which the uniqueness of each salt marsh depends and contribute substantially to increasing the biodiversity of these specialised habitats [32]. The coastal marshes near the city of Valencia in eastern Spain (as those in La Albufera Natural Park) shelter a large number of these species of great ecological and conservation value [32,40]. Most of these species have not been previously studied, and their limits and mechanisms of stress tolerance are virtually unknown [32,34,41].

From the point of view of its conservation, as is the case for *L. maritinum*, only the germination capacity of its seeds has been studied [42] and their collection for ex situ conservation (e.g., in the Valencian “Centre for Forestry Research and Experimentation” (CIEF)—Wildlife Service, Generalitat Valenciana). Work related to its physiology is essential for successful future translocation actions in the natural environment.

The present work aims to comprehensively characterise the biochemical responses of *L. maritimum* to salt stress, with a focus on identifying salt tolerance mechanisms and species-specific adaptations and exploring the intricate relationship between salt stress and the flowering, fructification and further germination of seeds produced by the salt-treated plants. By doing so, it seeks to inform practical conservation strategies and contribute to the broader understanding of ecological resilience in salt-affected habitats, ultimately safeguarding the survival of these coastal plant species and their ecosystems.

## 2. Results

### 2.1. Substrate Analysis and Growth Parameters

*Linum maritimum* plants were watered weekly with salt solutions of 50, 100, 200 and 300 mM of NaCl, and those from the control treatment with tap water. After six months of treatments, the electric conductivity of the substrate (EC_1:5_) increased considerably in parallel to the salt concentrations applied (Table 1). Only plants from the control, 50 and part of the 100 mM NaCl treatment groups survived, whereas those from the 200 mM NaCl treatment group died after 14 weeks, and those from the 300 mM NaCl treatment group died after 12 weeks (Table 1).

**Figure 1:**
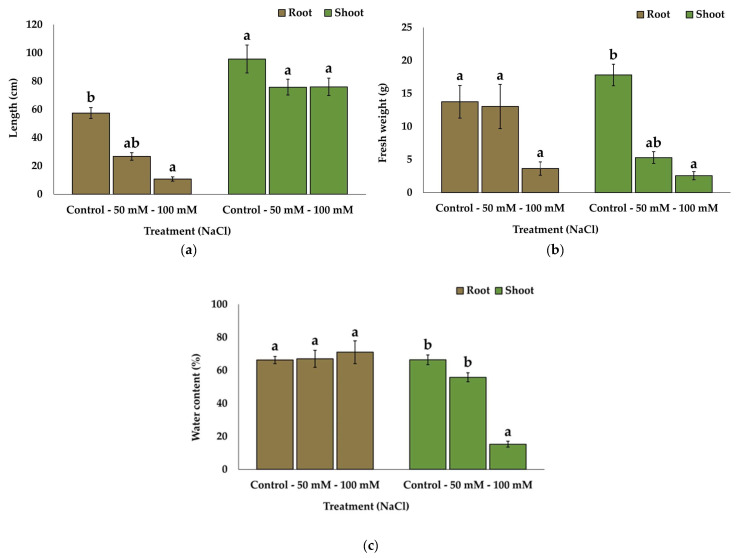
Length (**a**), fresh weight (**b**) and water content (**c**) of the roots and shoots of *Linum maritimum* after six months of salt treatments. Values show means ± SE (n = 5). The same letters indicate homogeneous groups between treatments for roots and for shoots, respectively, according to the Tukey test (*p* < 0.05).

Several vegetative growth parameters were analysed at the end of the treatments when the plant material was sampled. The 100 mM NaCl treatment strongly affected the length of the roots, as seen in Figure 1a. The roots of the plants subjected to this treatment had an average length of ca. 10 cm, whereas those in the control group had an average length of ca. 57 cm. There were no significant differences in shoot length between the different treatment groups, with the plants varying between 80 and 100 cm in height.

The fresh weight (Figure 1b) of both the roots and shoots decreased significantly under the 100 mM NaCl treatment, by ca. 65% in the roots and more than 80% in the shoots, compared to the control values.

The water content of the roots (Figure 1c) did not vary under the salt stress and remained stable at 67%, whereas in the shoots, it showed a significant reduction to ca. 15% in the presence of 100 mM of NaCl.

### 2.2. Reproductive Success

**Figure 2:**
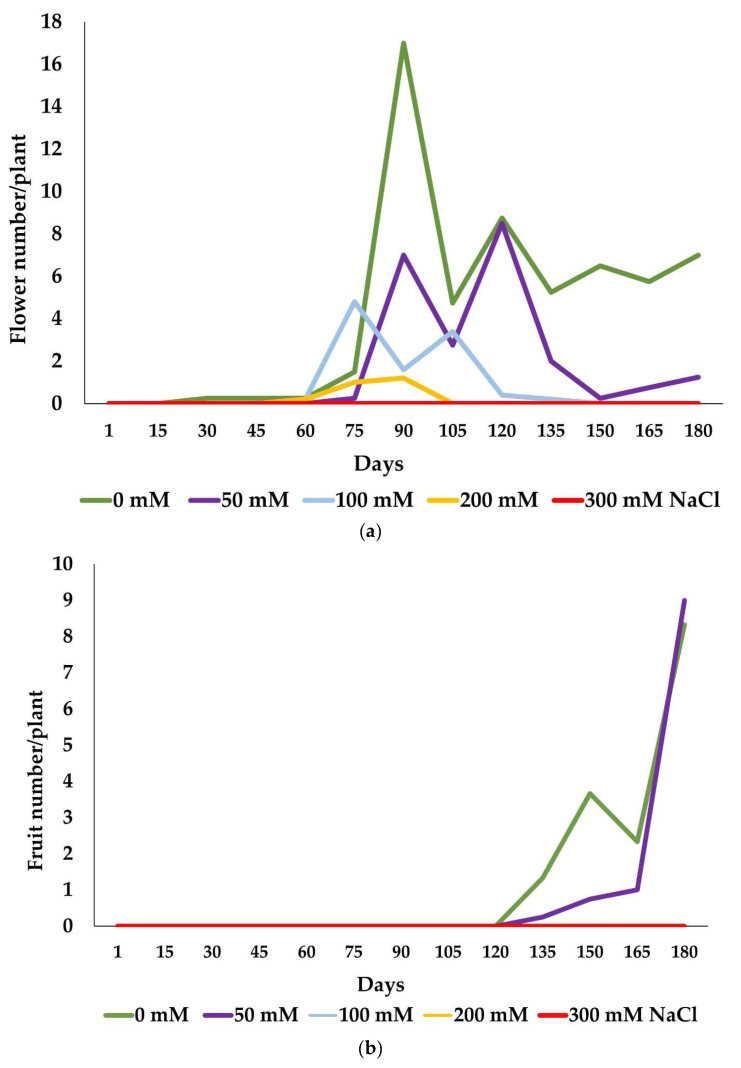
Evolution of flowering (**a**) and fruit set (**b**) showing the mean number of flowers and fruits per plant during the salt stress treatments in the spring and summer of 2022.

During the salt stress treatment, the highest number of flowers were formed in the control plants, followed by the 50 mM-NaCl-treated plants. Most of the flowers in the control group were produced in June 2022, but the plants subjected to the 50 mM NaCl treatment showed two flowering peaks, one at the beginning of June and the other in early July (Figure 2a). The plants treated with 100 mM NaCl started to bloom earlier than those in the control group, in mid-May, and had the highest flower production during early June, together with the 50 mM NaCl treatment group. The flowering decreased with increasing NaCl concentrations, and at 200 mM, there were only 11 flowers per plant, most of which were produced in late May. The plants subjected to the highest salt concentration (300 mM NaCl) did not produce flowers at all. Fruits were produced only by plants in the control and 50 mM NaCl treatment groups (Figure 2b); the fruit set started at the beginning of July and registered a peak during early September 2022, with eight and nine fruits per plant, respectively, in the two treatment groups.

Table 2 shows the effects of the different NaCl concentrations on the average number of flowers and fruits produced by the plants during the six months of treatments. The number of flowers per plant was reduced considerably with increasing salt concentrations. The control group produced the highest number of flowers, ca. 146 per plant, followed by a considerable decrease in the 50 mM NaCl (51 per plant) treatment group and stronger reductions in the presence of 100 mM and 200 mM of NaCl. The highest salt concentration tested, 300 mM of NaCl, completely inhibited flower development.

The number of fruits produced by the plants was significantly lower than of the number of flowers, which could be partly explained by the lack of pollinators. The fruit set was significantly reduced in the plants treated with 50 mM of NaCl compared with the controls, indicating that even low salinity levels had a negative impact on fruit formation. No fruits were produced in the plants subjected to higher salinities, 100 mM, 200 mM or 300 mM NaCl, revealing the susceptibility of *L. maritimum*’s reproductive development to prolonged salinity.

**Figure 3:**
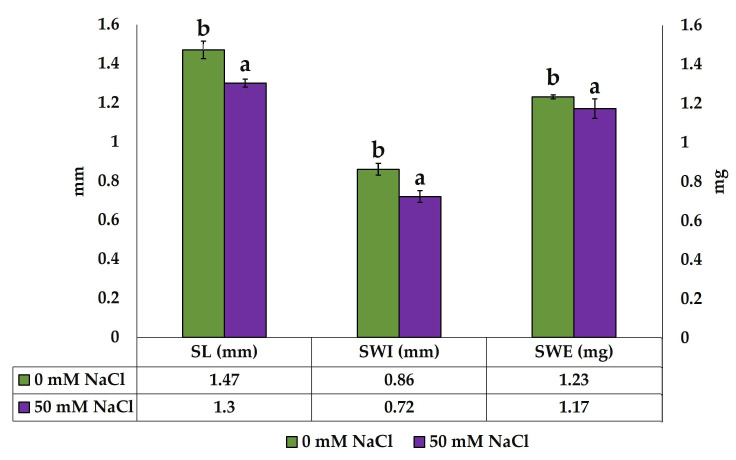
Mean seed length (SL), seed width (SWI) and seed weight (SWE) of seeds produced by control and 50 mM-NaCl-treated plants. Values shown are means per plate ± SE; n = 5. Different lowercase letters indicate significant differences between treatments for each determined variable, according to the Tukey test (*p* < 0.05).

The number of seeds in each fruit was uniform (10 seeds/fruit). The seeds produced by the control plants were slightly but significantly larger than those of the salt-treated plants in terms of length, width and weight (Figure 3).

### 2.3. Germination of Seeds Produced by Control and Salt-Treated Plants

The seeds from the harvested plants were subjected to germination tests in distilled water and 50 mM NaCl. Although smaller, the seeds produced by the salt-treated plants had a germination capacity similar to those from the control plants. The only significant difference in the germination percentages was found between the seeds from the control and NaCl-treated plants germinated in water, 52% and ∼70%, respectively. For all the other determined germination parameters, no significant differences were found, in general, between the seeds collected from the control and the salt-treated plants; however, most of those parameters were negatively affected by germination in the presence of salt (Table 3).

The mean germination time (MGT) was significantly higher in the seeds that germinated in 50 mM of NaCl, regardless of their origin. Also, the seeds produced by the plants grown in the presence of salt germinated earlier in distilled water, as their first germination day (FGD) was, on average, 2.8, which is significantly lower than the value determined for the seeds that germinated in 50 mM NaCl solution, 4.4. The seeds from control plants, on the contrary, started germinating at the same time in water and 50 mM NaCl. The presence of salt delayed seed germination, as the last germination day (LGD) and the total spread of germination (TSG) showed higher values in 50 mM NaCl than in water; for example, germination stopped on around day 15 in distilled water and on around day 26 in 50 mM NaCl, irrespective of the seed origin.

The germination index (GI) values were consistent in all the germination tests without significant differences. The GI is relevant for detecting the quality of seeds and their ability to develop into healthy plants, as it combines both the germination percentage and speed. A higher GI shows a larger percentage of seeds germinating successfully, implying better seed quality. The mean values of the speed of emergence (SE) also increased in the seeds germinating in the presence of salt compared to those germinating in water, which could be related to the salt-induced delayed germination; however, the observed differences were not statistically significant.

For seedling parameters, the seedling vigour index (SVI) showed a decreasing trend when germinating the seeds in the presence of salt, but the differences with the control seeds were not statistically significant. The SVI of the seeds collected from the salt-treated plants was slightly higher than that of the seeds from the non-stressed plants, both in the presence and absence of NaCl in the germination medium; however, here again, the differences were not significant (Table 3). A similar pattern of variation was detected for the hypocotyl length measured at 14 days, which ranged from a minimum of 2.22 to a maximum of 4.24 mm in the same treatments. In contrast, the radicle length was significantly shorter (less than 5 mm) only in the seeds produced by the water-irrigated plants that germinated in the presence of NaCl, while in all the other conditions, the mean values were higher than 9 mm (Table 3). The same pattern of variation was found for the mean seedling length, with a value of 7.21 mm in the same seeds, significantly different from all the others.

The analysis of germination was completed with a two-way ANOVA, considering two factors, the growth conditions of the plants that produced the seeds (A) and the germination conditions (B), and their interaction (A × B), as shown in Table 4. Factor A (the plant growth conditions) had a significant effect only on the seed size, with the seeds produced by the plants irrigated with water having a greater length and width. The germination conditions (factor B) significantly influenced some parameters related to the germination time (MGT, LGD, TSG), as in the presence of salts, the seeds took longer to germinate. The hypocotyl length and seedling lengths were also affected by this factor, but not the radicle length or seedling vigour (SVI).

**Figure 4:**
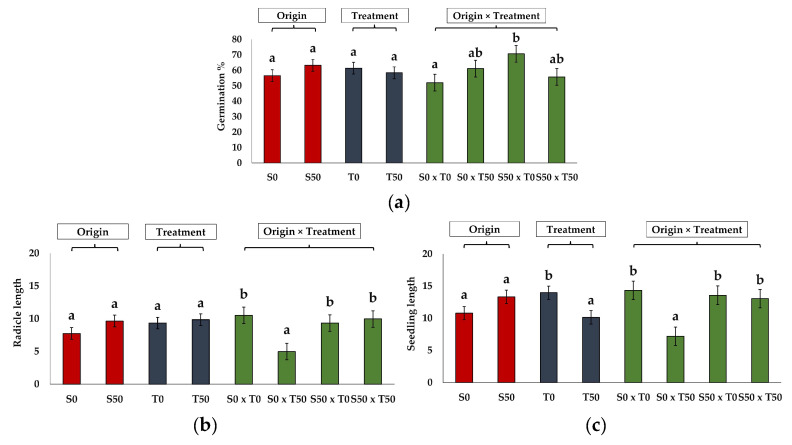
The unilateral effect of the source of seeds (origin), the conditions of germination (treatment) (**b**), and the combined effect of the two factors for parameters that showed significant interaction with the two factors: germination percentages (**a**), mean radicle length (**b**), and mean seedling length (**c**). Significant differences between means are illustrated within each treatment with different letters, according to the Tukey test (*p* < 0.05). Abbreviations: S0, seeds produced by control plants grown without salt; S50, seeds produced by plants treated with 50 mM NaCl for six months; T0, germination in distilled water; and T50, germination in 50 mM NaCl solution.

The interactions of the two factors, A and B, were only significant for the germination percentage (Figure 4a), radicle length (Figure 4b) and seedling length (Figure 4c). Although neither origin nor treatment had a significant effect on the seed germination, the interaction of the two factors indicates that the two sources of seeds responded differently, with a peak of germination in the seeds produced by the salt-treated plants that germinated in distilled water and the lowest value for the seeds from the control, non-stressed plants that also germinated in water. Furthermore, in the case of the radicle and seedling lengths, only the interaction of the two factors was significant, but not their effect when analysed separately. The seeds produced by the non-stressed plants that germinated in 50 mM NaCl were the worst performing, whereas all the others showed a similar response.

### 2.4. Analysis of Biochemical Parameters

The results of the biochemical analyses carried out in the plants sampled after six months of salt treatment are summarised in Table 5. Only the plants from the control and 50 mM NaCl treatment groups were used for the biochemical analysis; the plants from the 200 and 300 mM NaCl treatment groups did not survive, and the plant material from the 100 mM treatment group was insufficient because some of the plants from this treatment group also perished before the material was harvested.

Decreases in chlorophyll a and b and carotenoids were found in the plants from the salt treatment, although these differences were not statistically significant.

The mean proline concentration did not rise significantly in response to the salt stress; it reached 1.5 µmol g^−1^ DW in the presence of 50 mM NaCl compared to 1.3 µmol g^−1^ DW in the control plants. The plants grown under the control conditions and those watered with 50 mM NaCl did not exhibit a significant difference in their total soluble sugar (TSS) leaf contents. On the contrary, the content of the other quantified osmolyte, glycine betaine, increased significantly from 33.2 µmol g^−1^ DW in the control plants to 93.2 µmol g^−1^ DW in the plants treated with 50 mM of NaCl, supporting the compound’s contribution to osmotic adjustment under stress in *L. maritimum*.

The malondialdehyde (MDA) concentrations also showed significant differences; the plants treated with 50 mM NaCl accumulated three-fold as much MDA in their leaves as the control group, which indicates a salt-induced generation of oxidative stress. MDA is a product of lipid peroxidation and is considered a reliable marker of oxidative stress. This result was supported by a parallel increase of about 3.3-fold in the contents of another oxidative stress biomarker, hydrogen peroxide (H_2_O_2_).

The total phenolics (TPCs) and flavonoids (TFs), two representative groups of antioxidant metabolites, also increased significantly—about two- and three-fold, respectively—in the salt-treated plants with respect to the controls.

### 2.5. Ion Accumulation

**Figure 5:**
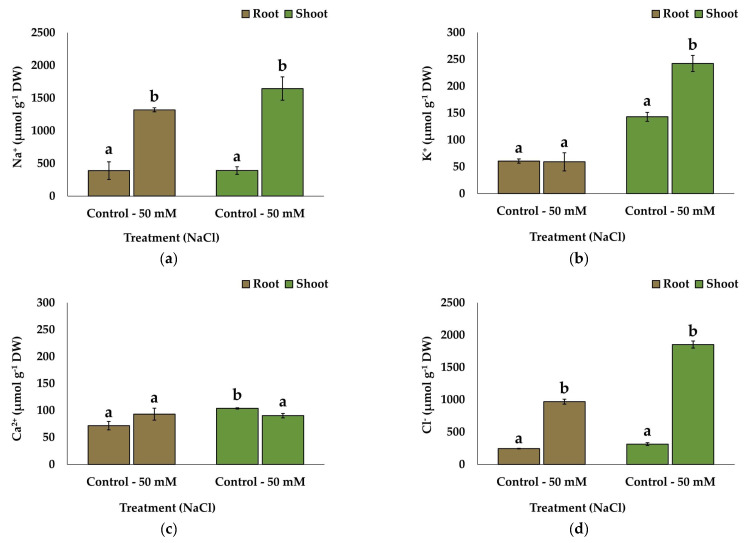
Effect of stress treatments on the root and shoot contents of ions: (**a**) sodium; (**b**) potassium; (**c**) calcium; and (**d**) chloride. Values shown are means ± SE; n = 5. The same letters indicate homogeneous groups between treatments for roots and for shoots, respectively, according to the Tukey test (*p* < 0.05).

The Na^+^ level increased significantly, 3.3-fold in the roots and 4.2-fold in the shoots, in the salt-stressed plants with respect to the controls. These increases indicate a substantial accumulation of Na^+^ ions under salt stress, reaching over 1.5 mmol g^−1^ DW in the shoots (Figure 5a), a common reaction in plants exposed to saline environments.

The K^+^ concentration in the roots remained steady, at around 60 µmol g^−1^ DW in the control and salt-treated plants, whereas watering the plants with 50 mM of NaCl led to the accumulation of K^+^ in the shoots reaching almost double the levels in the non-stressed control plants (Figure 5b). This finding suggests that potassium ions are actively translocated to the shoots to counteract the effects of the accumulation of toxic Na^+^ ions.

Regarding the divalent cation Ca^2+^, it accumulated to higher levels in the shoots than in the roots of the non-stressed plants. The salt treatment caused a slight, statistically non-significant increase in the Ca^2+^ contents in the roots and a slight but significant decrease in the shoots (Figure 5c).

Finally, the anion Cl^−^ concentrations changed following the same pattern as Na^+^, increasing in response to the salt treatment in the roots and shoots, ca. 3.9- and 6-fold, respectively (Figure 5d).

## 3. Discussion

The results presented here reveal that *Linum maritimum*, although considered a halophyte and included in the database of halophytes “eHALOPH” [43], is susceptible to prolonged exposure to high NaCl concentrations. The salt tolerance limits of halophytes are highly variable, as this category includes species belonging to about 40 genera in different families [44] from a wide variety of habitats, ranging from mangroves, coastal and inland marshes, dunes and cliffs to semi-deserts and alkaline deserts. Early definitions in the 19th century referred to this group of species simply as plants growing on saline soils in littoral zones or salt lakes [45]. Later, different ecological and physiological criteria were used to characterise this category; however, the most widely accepted is the operational definition [4] that considers halophytes as plants able to survive and complete their life cycle in habitats with a soil salinity equivalent to, at least, 200 mM of NaCl.

In the Western Mediterranean, *L. maritimum* occurs mainly in communities of the phytosociological class *Juncetalia maritima*, included as habitat type “1410 Mediterranean salt meadows” in the Habitats Directive [46]. In the Iberian Peninsula, the species has been reported in several coastal and interior communities, although it is most commonly found in the association of *Schoeno nigricantis–Plantaginetum crassifoliae* Br.-Bl. ex Tomaselli 1947, where it is a characteristic species [47]. This type of community develops on clayey, basic, slightly carbonated soils, rich in organic matter, brackish, close to salt marshes and in depressed and frequently humid places [47]. A report on soil salinity in the Valencia area indicates that the electric conductivity values in saturation aqueous extracts (ECe) are around 22 dS m^−1^ [48]. This is in the range of the values recorded in our experimental conditions after six months of irrigation with 50 mM of NaCl using a conversion factor [49] suitable for the sandy loam texture of soils such as those typically supporting this plant community [50].

The high salinity of the substrates reached in the salt stress treatments had a negative effect on the plants. The 300 mM NaCl solution, with the highest concentration of salt applied, inhibited flowering completely and was lethal after three months. The plants from the 200 mM NaCl treatment group produced a few flowers, which did not develop further into fruits. After three and half months, all the plants from this treatment perished. On the contrary, most of the plants (60%) watered with 100 mM NaCl survived the six months of treatment, but their growth was severely affected. An advance in floral phenology was observed with the plants from this treatment, which showed a first blooming peak more than two weeks earlier than the others that reached flowering. Halophytes generally show delayed and more extended flowering under optimal salinity [51,52], whereas early flowering and floral abortion under saline stress are responses typical of glycophytes [52]. As such, the concentration of 100 mM NaCl is beyond the optimal level in this species, as also proved by the absence of a fruit set in this treatment group. All the plants irrigated with the 50 mM NaCl solution survived during the six months of treatment, and only the fresh weight of the shoots was significantly lower than that of the control. Flowers and fruits were produced, albeit in a smaller number than in the non-stressed control group, where the maximum values of reproductive traits were registered. Although the best plant performance was found in the control group, the final EC in this treatment group indicated that a slight salinisation occurred due to the accumulation of salts in the tap water used to irrigate the plants. However, in this treatment group, the number of fruits was much lower than the number of flowers, which is probably related to the lack of pollinators in the closed greenhouse environment. *Linum maritimum* is distylous [53] and, like other species of this genus with this floral trait, self-incompatible [54], so cross-pollination is necessary.

Seeds were only produced in the control and the 50 mM-NaCl-treated plants, and their size was slightly but significantly smaller in the latter. However, their germination capacity and seedling vigour were similar when they germinated in distilled water. The germination under 50 mM of NaCl was delayed, as reflected by several calculated indexes, independently of the conditions of the growth of the maternal plants; however, better seedling performance in terms of radicle and total lengths was observed in those resulting from the salt-treated plants. A positive effect of the exposure of the maternal plants to salinity on the germination rate and seedling performances was also reported in *Iris hexagona* [55]. Maternal salinity is found to improve the yield, size and stress tolerance of *Suaeda fruticosa* seeds [56]. Similarly, in the facultative desert halophyte *Zygophyllum coccineum*, better germination, recovery of germination, and seedling development were found in plants from a saline environment than in those from a non-saline area, indicating salt tolerance derived from maternal exposure to salinity [57]. On the contrary, for typical glycophytes, the growth of mother plants under saline conditions generally has a negative effect on their seeds, as reported in different crops and ornamental plants [58,59,60].

The study was completed by determining several biochemical stress markers in plant material from two treatments (control and 50 mM of NaCl), the only ones that provided sufficient samples to be analysed. This is the first study of this kind on *L. maritimum*, as abiotic stress responses have been investigated virtually in only one species of this genus, *L. usitatissimum* (flax), which is of great importance as an industrial crop and medicinal plant. Although the EC of the substrate in the 50 mM NaCl treatment group was more than double that in the control plants, the photosynthetic pigment concentrations did not vary significantly between the two treatments. On the contrary, most reports on flax showed a significant reduction under salt stress [61,62] due to the inhibition of chlorophyll synthesis and the activation of its degradation with increased salinity [63].

Proline, a common osmolyte in plants that increases in response to various forms of abiotic stress, is another suitable stress biomarker, in addition to photosynthetic pigment degradation [64]. Proline is one of the most common osmolytes in plants, which, apart from its role in osmotic adjustment, plays additional functions as an osmoprotectant, stabilising subcellular structures and macromolecules such as membranes and proteins, scavenging free radicals, and functioning as a signalling molecule in stress responses [65,66]. Several reports show a significant increase in proline in flax under salt stress [62,67,68], some of them indicating that the more stress-tolerant genotypes accumulate higher Pro concentrations [69,70], although the opposite has also been reported [53].

In *L. maritimum*, only low levels of Pro were detected in comparison to those reported in flaxseed [62], but another common osmolyte in plants, glycine betaine, was present in higher concentrations and showed a significant variation in response to the change in the substrate salinity, as also reported in flax [71] and *Linum album*, an endemic from Iran with medicinal properties [72]. However, in this latter species, the reported GB concentrations were much higher than in *L. maritium* or *L. usitatissimum* and similar to those measured in plants that are typical glycine betaine accumulators [73]. GB is a quaternary ammonium compound that, in addition to its function as a compatible solute, could be involved in inhibiting ROS accumulation, protecting membranes and the photosynthetic machinery and activating some stress-related genes. GB has also been associated with protecting the quaternary structure of proteins (thus maintaining enzymatic activity) from the damaging effects of environmental stresses [74]. Furthermore, it has been reported that GB could also affect K^+^ efflux by regulating ion channels [75].

The concentrations of total soluble sugars measured in our experimental conditions were similar to those published in flax, although they did not vary significantly, as found in this species [71]. TSSs have been reported to play a specific function as osmolytes and osmoprotectants in stress tolerance mechanisms in different species (e.g., [76]). However, their role in osmoregulation under stress is more difficult to assess due to their multiple biological functions as direct products of photosynthesis, metabolic precursors and energy sources, and, therefore, they are involved in many physiological processes.

Reactive oxygen species (ROS) are produced in greater quantities in response to abiotic stress. Originally thought to be just harmful byproducts of aerial metabolism that led to oxidative stress, ROS are now well recognised for their crucial role as signalling messengers in several essential physiological processes [77]. Malondialdehyde (MDA), a product of membrane lipid peroxidation, and hydrogen peroxide (H_2_O_2_) are frequently used to assess the degree of oxidative stress that plants experience and their susceptibility to a specific type of stress (e.g., [78]). These markers significantly increased in the salt-treated *L. maritimum* plants, indicating that these plants suffer from salinity-associated oxidative stress. Plants employ two primary defence mechanisms against harmful ROS levels: the production of low-molecular-weight antioxidant compounds, such as ascorbic acid (AA) or phenolic compounds, particularly the subgroup of flavonoids, and a series of antioxidant enzymes [79]. Although it has been documented that the concentrations of phenolics and flavonoids rise in many halophytes in response to stress [80], no significant variation has been noted in many other more salt-tolerant species, which most likely have effective strategies to prevent excessive ROS generation [81]. In *L. maritimum*, the total phenolics and flavonoids increased significantly in the plants subjected to salt treatments, as reported in flax [68]. High amounts of alpha-linolenic acid and flavonoids were reported in linseeds, making *L. usitatissimum* an interesting source of nutraceutical compounds with beneficial effects on human health [82].

Finally, the analysis of the different ion concentrations in the roots and shoots of the salt-treated plants revealed an interesting pattern, relevant to *L. maritimum*’s salt tolerance mechanisms: the Na^+^ accumulation in the shoots was not accompanied by a decrease in K^+^, as observed in flax [62,68,83] and many other species. K^+^ is an essential nutrient and the most abundant plant cation [84]. K^+^ levels often drop as Na^+^ accumulates because both cations compete for the same protein transporters [85]. Furthermore, Na^+^ activates outward rectifying K^+^ channels, which results in plasma membrane depolarisation and additional cellular K^+^ loss [79,86]. Therefore, an increase in Na^+^ contents generally results in a parallel reduction in K^+^ concentrations. However, many halophytes share mechanisms that enable the maintenance of high leaf K^+^ contents in the presence of salt [87] and even the activation of K^+^ transport to shoots at high soil salinities, as reported for some other halophytes from the same area as *L. maritimum* [88,89,90].

## 4. Materials and Methods

### 4.1. Plant Growth and Stress Treatments in the Greenhouse

Adult plants of *L. maritimum* were provided by the Centre for Forestry Research and Experimentation (CIEF)**,** Valencia, Spain, on 17 February 2022, in pots of 12 cm diameter and 17 cm height, filled with a mixture of peat, perlite and coco fibre (4:1:1). The plants were obtained by the germination of seeds from the Germplasm Bank of the Wildlife Service and the Natura 2000 network of Generalitat Valenciana (reference 2213V3A4), sampled in La Albufera Natural Park (Valencia, Spain). During the acclimation period, the plants were watered twice a week with tap water, and then the treatments were started on 10 March 2022, when the first flower buds appeared.

The pots were placed on five plastic trays (55 cm × 40 cm), and the following treatments were applied: control plants grown in the absence of salt (C) and four salt treatments (50 mM, 100 mM, 200 mM and 300 mM NaCl). The number of replicates was five individual plants in each treatment. From 10 March 2022 to 20 May 2022, the plants were watered twice a week with 1.5 L of water (control) or NaCl solutions at the concentrations mentioned above; from 23 May 2022 to 5 September 2022, the irrigation volume was increased to 2 L, and the plants were watered three times a week, twice as before, with water or the different salt solutions, and the third time using only water for all the plants. The treatments were stopped after six months, when the fruits and seeds were formed.

### 4.2. Substrate Analysis

Substrate electroconductivity (EC) evolution was controlled weekly with a WET-2 Sensor (Delta—T Devices, Cambridge, UK). At the end of the treatments, the electrical conductivity (EC 1:5) was measured in the laboratory with a Crison 522 conductivity meter (Crison Instruments SA, Barcelona, Spain).

### 4.3. Plant Growth and Reproductive Parameters

The number of flowers and fruits was determined weekly during the treatments. The fresh weights of the shoots and roots were separately measured after six months of treatment when all plants were sampled. Part of the fresh material of each organ was weighed (fresh weight; FW), dried for five days at 65 °C until it reached constant weight, and then weighed again (dry weight; DW) to calculate the water content percentage (WC%), according to the following formula:

WC% = [(FW − DW)/FW] × 100.(1)

The samples of fresh plant material (0.05–0.15 g) were frozen in liquid N_2_ and stored at −75 °C in properly labelled 2 mL Eppendorf tubes until they were used for the biochemical analyses. The samples of dry material were kept in paper bags at room temperature.

### 4.4. Seed Germination Tests

In the harvesting phase, the number of seeds was counted for all the fruits of all the plants before being used for the germination tests. The germination capacity of the seeds produced by the non-stressed control plants and those treated with 50 mM of NaCl was checked in distilled water and in the presence of 50 mM of NaCl.

The seeds were placed in standard Petri dishes with a 55 mm diameter on a double layer of filter paper moistened with 1.5 mL of solution. The plates were kept in an Equitec germination chamber, configured with a daytime temperature of 30 °C for 16 h and a nighttime temperature of 20 °C for 8 h. Seeds with at least 1 mm radicle protrusions were considered germinated.

The number of germinated seeds was registered daily over 30 days. The germination capacity was expressed as the percentage of germination (GP) and the germination rate as the mean germination time (MGT), which was calculated according to the formula:

MGT = ∑Dn/∑n(2)

where D represents the number of days from the beginning of the germination test, and n is the number of seeds newly germinated on day D [91].

After fourteen days of germination, the lengths of the radicles and hypocotyls of the germinated seeds were measured and analysed using Digimizer v.4.6.1 software (MedCalc Software, Ostend, Belgium, 2005–2016). The following additional indexes were then determined:

The germination index (GI), which is a strong indicator of the success and speed of germination [92], was calculated using the equation:

GI = ∑G/T(3)

where G is the number of germinated seeds on a specific day, and T is the number of days from the start of the experiment until that day.

The speed of emergence (SE) [93], to determine the germinative energy through the germination speed, was calculated using the equation:

SE = [(number of germinated seeds on the first day of germination)/(number of  germinated seeds on the last day of germination)] × 100(4)

And the seedling vigour index (SVI) was calculated using the equation described in [94]:

SVI = (Seedling length, in mm × Germination percentage)/100(5)

The number of germinated seeds was registered daily for 30 days.

### 4.5. Biochemical Analyses

#### 4.5.1. Photosynthetic Pigments

Photosynthetic pigments were extracted from samples of the ground fresh shoot material (ca. 0.05 g) with 1 mL of ice-cold 80% acetone (*v*/*v*) by mixing in a rocker shaker for 24 h in darkness. The samples were centrifuged at 13,300× *g* for 10 min at 4 °C. The supernatant was diluted 10-fold with 80% acetone, and the absorbance was measured at 470 nm, 646 nm and 663 nm. The concentrations of chlorophyll a (Chl a), chlorophyll b (Chl b) and carotenoids (Caro) were calculated according to Lichtenthaler and Wellburn [95] and expressed in mg g^−1^ DW.

#### 4.5.2. Quantification of Osmolytes

The quantification of proline (Pro) was carried out according to Bates et al. [96]. Samples of the ground fresh shoot material (ca. 0.5 g) were extracted in 0.5 mL of a 3% (*w*/*v*) aqueous sulphosalicylic acid solution and mixed with 0.5 mL of acid ninhydrin. The samples were incubated in a water bath for 1 h at 95 °C, cooled on ice for 10 min, and then extracted with 3 mL of toluene. The absorbance of the organic phase was measured at 520 nm using toluene as the blank. Samples with known Pro concentrations were assayed in parallel to obtain a standard curve. The proline contents were expressed as µmol g^−1^ DW.

The total soluble sugars (TSSs) were determined following the method of Dubois et al. [97]. Samples of 0.05 g of fresh ground material were extracted overnight with 80% (*v*/*v*) methanol, and the supernatant obtained upon centrifugation was mixed with 5% phenol and concentrated sulphuric acid. Spectrophotometric measurements of the solutions were then performed at 490 nm. The TSS concentrations were expressed as equivalents of glucose, which was used as the standard (mg eq. glucose g^−1^ DW).

The glycine betaine (GB) concentration was determined as described by Grieve and Grattan [98] with some modifications [99]. The fresh shoot material (0.15 g) was shaken for 24 h at 4 °C with 1.5 mL of Mili Q water and then centrifuged at 13,300× *g* for 10 min. The supernatant was mixed (1:1) with a 2 N H_2_SO_4_ solution and stored in ice for 1 h. Then, 50 µL of ice-cold KI-I_2_ solution was added to 125 µL of the sample, inducing glycine betaine precipitation in the form of dark golden crystals. The samples were maintained at 4 °C for 16 h in darkness and then centrifuged for 45 min at 0 °C. The supernatant was gently removed, and the glycine betaine crystals were dissolved into 1.4 mL of cold 1,2-dichloroethane. The tubes were kept for 2.5 h under dark and cold conditions, and, finally, their absorbance was recorded at 365 nm. The glycine betaine concentration was calculated with a GB standard calibration curve and expressed as µmol g^−1^ DW.

#### 4.5.3. Determination of Oxidative Stress Markers and Antioxidant Compounds

For the determination of the concentrations of malondialdehyde (MDA), total flavonoids (TF) and total phenolic compounds (TPC), methanol extracts were prepared as for the total soluble sugars (TSS) measurements. Ground fresh shoot material (0.05–0.10 g) was extracted with 2 mL of 80% methanol, and the samples were centrifuged at 13,300× *g* and 4 °C for 15 min. The supernatants were transferred to fresh Eppendorf tubes and stored at −20 °C.

The MDA quantification followed a published procedure (Hodges et al. [100]); the methanol extracts were mixed with 0.5% thiobarbituric acid (TBA) in 20% trichloroacetic acid (TCA)—or with 20% TCA without TBA for the controls—and then incubated for 15 min at 95 °C in a water bath. The reactions were stopped on ice, and the samples were centrifuged at 13,300× *g* for 10 min at 4 °C. Finally, the absorbance of the supernatants was measured at 532 nm. After subtracting the non-specific absorbance at 600 and 440 nm, the MDA concentrations were calculated by applying the equations described by Taulavori et al. [101], based on the molar extinction coefficient of the MDA–TBA adduct at 532 nm (ε_532_ = 155 mM^−1^ cm^−1^).

The amount of hydrogen peroxide (H_2_O_2_), another oxidative stress marker, was measured according to Loreto and Velikova [102]. Fresh shoot material (0.05 g) was extracted with a 0.1% (*w*/*v*) trichloroacetic acid (TCA) solution and then centrifuged for 15 min at 4 °C. An aliquot of 500 µL of supernatant was mixed with 500 µL of 10 mM potassium phosphate buffer (pH 7) and 1 mL of 1 M potassium iodide. The absorbance was determined at 390 nm, and a standard curve was obtained from samples containing known H_2_O_2_ concentrations. The H_2_O_2_ contents were expressed as µmol g^−1^ DW.

The TPC quantification was performed by reacting the methanolic extracts with the Folin–Ciocalteu reagent in the presence of Na_2_CO_3_ [103]. The reaction mixtures were incubated for 90 min at room temperature in the dark, and the absorbance was measured at 765 nm. Gallic acid (GA) was used as the standard, and the TPC concentrations were expressed as GA equivalents (mg eq. GA g^−1^ DW).

The TF determination was carried out according to the method published by Zhishen et al. [104], based on the nitration with NaNO_2_ of aromatic compounds containing a catechol group, followed by a reaction with AlCl_3_ at a basic pH. After the reaction, the absorbance of the sample was measured at 510 nm, and the TF concentrations were expressed as equivalents of catechin, which was used as the standard to obtain a calibration curve (mg eq. C g^−1^ DW).

### 4.6. Quantification of Ions

The concentrations of sodium (Na^+^), potassium (K^+^), calcium (Ca^2+^) and the anion chloride (Cl^−^) were determined separately in the roots and shoots following the method described by Weimberg [105]. Samples of 0.1 g of ground dry material were extracted in boiling Milli-Q water (Merck, Rahway, NJ, USA), cooled on ice and filtered through a 0.45 µm Gelman nylon filter (Pall Corporation, Port Washington, NY, USA). The cations were quantified with a PFP7 flame photometer (Jenway Inc., Burlington, VT, USA), and the Cl^−^ concentration was measured using a chlorimeter, Sherwood 926 (Cambridge, UK).

### 4.7. Statistical Analysis

The statistical analyses of the data were performed using SPSS Statistics statistical software v. 16 (IBM SPSS Statistics) and Statgraphics Centurion XVI (Statgraphics Technologies, The Plains, VA, USA).

An analysis of variance (one-way ANOVA) was used to estimate the effects of the stress treatments on the traits analysed. The Tukey test was used as a post hoc test at a *p*-value of 0.05 (*p* < 0.05) to analyse the differences if the null hypothesis was rejected.

A two-way ANOVA was applied to check the effect of the origin of seeds in addition to that of the treatment and the significance of the interaction of the two factors.

## 5. Conclusions

*Linum maritimum* can be considered a halophyte, as it grows on light-to-medium saline soils in its natural habitats. It also possesses some stress tolerance mechanisms characteristic of many halophytes, such as increased K^+^ uptake and the activation of its transport from the roots to the shoots under salinity stress. The germination capacity of the seeds produced by the plants subjected to a long-term salt treatment was not impaired; the seedling performance under the low salt concentrations was even enhanced. However, our study revealed that the species is susceptible to high salinity, primarily affecting its reproductive capacity; prolonged exposure to salt stress is even lethal for the plants. This finding has implications for the conservation of the species, especially under changing climatic conditions that may lead to increased soil salinity in its Mediterranean distribution area. Therefore, the population reinforcement strategy should take into account the location with the lowest risk of salinization. On the other hand, facing future scenarios, most coastal halophytes, such as *L*. *maritimum*, could be threatened by sea level rise, one of the expected effects of climatic change, but many of their current habitats are impossible to recreate artificially. Similar future problems can be drafted for other coastal endangered species worldwide currently benefiting from translocation projects.

## Figures and Tables

**Table 1:** Electric conductivity of the substrate and survival of plants after six months of irrigation with NaCl solutions. Values shown are means ± SE; n = 5. Different lowercase letters indicate significant differences between treatments, according to the Tukey test (*p* < 0.05).

Treatment	Control	50 mM NaCl	100 mM NaCl	200 mM NaCl	300 mM NaCl
EC_1:5_ (dS m^−1^)	1.50 ± 0.2 a	3.37 ± 0.4 b	4.03 ± 0.7 b	9.04 ± 1.2 c	11.06 ± 1.5 c
Survival rate (%)	100	100	60	0	0

**Table 2:** Mean values of flowers and fruits per plant produced during the six months of salt treatments. Values shown are means ± SE; n = 5. Different lowercase letters indicate significant differences between treatments for each determined variable, according to the Tukey test (*p* < 0.05).

Treatment	Control	50 mM NaCl	100 mM NaCl	200 mM NaCl	300 mM NaCl
Flower number	146.65 ± 23.9 c	51.01 ± 1.5 b	39.20 ± 16.9 ab	19.80 ± 3.8 a	0
Fruit number	17.68 ± 2.6 a	11.50 ± 2.3 a	0	0	0

**Table 3:** Seed germination after 30 days of assay. Values shown are means per plate ± SE; n = 5. Different lowercase letters indicate significant differences between treatments for each determined variable, according to the Tukey test (*p* < 0.05). Abbreviations: GP, germination percentage; MGT, mean germination time; FGD, first germination day; LGD, last germination day; TSG, time spread of germination; GI, germination index; SE, speed of emergence; SVI: seedling vigour index, Hyp L, hypocotyl length; Rad L, radicle length.

Source of Seeds	Control		50 mM NaCl	
Germination Parameters	Control	50 mM NaCl	Control	50 mM NaCl
GP	52.00 ± 8.0 a	61.00 ± 5.1 ab	70.70 ± 11.0 b	55.70 ± 10.9 ab
MGT	7.95 ± 0.9 a	11.71 ± 1.2 b	7.83 ± 1.3 a	13.22 ± 1.1 b
FGD	4.00 ± 0.0 ab	4.00 ± 0.60 ab	2.80 ± 0.2 a	4.40 ± 0.5 b
LGD	14.8 ± 3.8 a	25.40 ± 1.0 b	15.00 ± 4.9 a	26.00 ± 0.5 b
TSG	10.80 ± 3.8 a	21.40 ± 1.5 b	12.20± 5.0 ab	21.60 ± 0.8 b
GI	0.87 ± 0.1 a	0.77 ± 0.2 a	0.99 ± 0.1 a	0.56 ± 0.1 a
SE	29.95 ± 3.8 a	39.33 ± 8.9 a	24.24 ± 3.3 a	31.67 ± 5.5 a
SVI	7.60 ± 1.5 ab	4.50 ± 0.7 a	9.70± 1.6 b	7.72 ± 2.7 ab
Hyp L (mm)	3.82 ± 0.5 a	2.22 ± 0.3 a	4.24 ± 0.3 b	3.10 ± 0.5 b
Rad L (mm)	10.52 ± 1.4 ab	4.99 ± 0.7 a	9.34 ± 0.9 b	9.96 ± 1.6 ab
Seedling length (mm)	14.35 ± 1.6 b	7.21 ± 0.9 a	13.59 ± 0.6 b	13.10 ± 2.0 b

**Table 4:** Two-way ANOVA (F values) considering the effects of the “origin of seeds”, “treatment” and their interactions on the germination parameters analysed. Abbreviations as in Table 3.

Parameter	Origin	Treatment	Origin × Treatment
GP	1.54 ^ns^	0.30 ^ns^	4.99 *
MGT	0.32 ^ns^	14.62 **	0.47 ^ns^
FGD	0.91 ^ns^	3.66 ^ns^	3.66 ^ns^
LGD	0.02 ^ns^	11.58 **	0.00 ^ns^
TSG	0.06 ^ns^	9.28 **	0.03 ^ns^
SVI	4.09 ^ns^	3.72 ^ns^	0.21 ^ns^
GI	0.13 ^ns^	3.07 ^ns^	1.04 ^ns^
SE	1.31 ^ns^	2.06 ^ns^	0.03 ^ns^
Hyp L	2.01 ^ns^	8.94 **	0.26 ^ns^
Rad L	2.26 ^ns^	3.79 ^ns^	5.95 *
Seedling length	3.10 ^ns^	7.00 *	5.24 *

*, ** significant at *p* = 0.05 and 0.01, respectively; ns: not significant.

**Table 5:** Effect of stress treatments on the shoot contents of photosynthetic pigments, osmolytes, oxidative stress biomarkers and antioxidant compounds: chlorophylls a and b (Chl a and Chl b), carotenoids (Caro), proline (Pro), total soluble sugars (TSS), glycine betaine (GB), malondialdehyde (MDA), hydrogen peroxide (H_2_O_2_), total phenolic compounds (TPCs) and total flavonoids (TFs). Values shown are means ± SE; n = 5. Different lowercase letters indicate significant differences between treatments for each determined variable, according to the Tukey test (*p* < 0.05). GA: gallic acid; C: catechin.

Treatment	Control	50 mM NaCl
Chl a (mg g^−1^ DW)	1.27 ± 0.2 a	0.99 ± 0.3 a
Chl b (mg g^−1^ DW)	0.63 ± 0.0 a	0.35 ± 0.2 a
Caro (mg g^−1^ DW)	0.34 ± 0.0 a	0.29 ± 0.0 a
Pro (µmol g^−1^ DW)	1.33 ± 0.6 a	1.56 ± 0.7 a
TSS (mg glucose g^−1^ DW)	17.62 ± 2.5 a	18.98 ± 1.7 a
GB (µmol g^−1^ DW)	33.21 ± 4.2 a	93.22 ± 12.8 b
MDA (nmol g^−1^ DW)	41.61 ± 5.2 a	127.67 ± 1.7 b
H_2_O_2_ (µmol g^−1^ DW)	1.61 ± 0.3 a	5.36 ± 0.3 b
TPC (mg eq. GA g^−1^ DW)	6.59 ± 0.4 a	13.61 ±1.3 b
TF (mg eq. C g^−1^ DW)	0.79 ± 0.2 a	2.33 ± 0.4 b

## Data Availability

Data are contained within the article.
